# Isolation and Aflatoxin B1-Degradation Characteristics of a *Microbacterium proteolyticum* B204 Strain from Bovine Faeces

**DOI:** 10.3390/toxins14080525

**Published:** 2022-07-30

**Authors:** Yi Yan, Xinyue Zhang, Haiyan Chen, Wenmin Huang, Hongnian Jiang, Chulun Wang, Zhuang Xiao, Yuyu Zhang, Jialiang Xu

**Affiliations:** School of Light Industry, Beijing Technology and Business University, Beijing 100048, China; yanyi@btbu.edu.cn (Y.Y.); zxyyue1998@sina.com (X.Z.); haiychen@sina.com (H.C.); amanda17806709955@hotmail.com (W.H.); hongnian_jiang@sina.com (H.J.); wcl20010424@sina.com (C.W.); clockwork1657@sina.cn (Z.X.); zhangyuyu@btbu.edu.cn (Y.Z.)

**Keywords:** AFB_1_, *Microbacterium proteolyticum*, degradation, detoxification

## Abstract

Aflatoxin B_1_ (AFB_1_) is one of the most harmful mycotoxins, raising serious global health and economic problems. Searching for biological approaches for effective and safe AFB_1_ degradation is imminent. In our study, *Microbacterium proteolyticum* B204 isolated from bovine faeces degraded 77% of AFB_1_ after 24 h, becoming the first reported bacteria from the Microbacterium family to possess AFB_1_ degradation characteristics. Temperature variation showed little effect on its degradation ratio, demonstrating high thermostability of 75% and 79% after boiling and sterilization, respectively. We suppose that the components playing a key role during this process were proteins, considering the decreased degradation rate caused by Proteinase K. Cell viability detection on HepG2 cells indicated that the degradation products were much less toxic than pure AFB_1_. Furthermore, B204 cell-free culture supernatant also degraded AFB_1_-contaminated food, such as peanuts, corn and cheese. These results suggested that this strain with AFB_1_ degradation properties could be a prospective candidate for application in the food and feed industries.

## 1. Introduction

Aflatoxins (AFs) are typical mycotoxins ubiquitous in agricultural and sideline commodities that are generated as highly toxic secondary metabolites produced mainly by *Aspergillus flavus* and *Aspergillus parasiticus* [1,2]. Among the four major isoforms of AFs—B1, B2, G1 and G2—AFB_1_ has been classified as a Class IA Danger and a Category I carcinogen by the WHO and IARC’s Human Carcinogen Risk Assessment Working Group due to its high toxicity [3,4,5]. AF contamination in tropical and subtropical regions becomes more severe, since fungi grow better under high temperatures and humidity. The harmful and toxic effects of AFs not only occur in food or feed, but also accumulate as food chains extend, which further spreads these effects [6,7]. As a result, AF contamination in food and the environment is an international concern on account of its tremendous hypertoxicity, mutagenicity, teratogenicity and carcinogenicity to both humans and livestock [2,8].

Searching for strategies to detoxify AFB_1_ safely and effectively therefore has become a hot topic of scientific studies; there is also an emergent demand for these strategies within national welfare and public health. The application of adsorbent products is one of the most commonly used physical methods to remove AF contamination, including heating and irradiation [9]. Acid, alkali and oxidizing agents (e.g., chlorine dioxide) are mainly used as chemical substances to disinfect toxins [10]. Moreover, active components, including curcumin, resveratrol and grape seed waste in the diet can also effectively alleviate AFB_1_ toxicity to animals [11,12]. However, the taste and appearance of food might be impaired after these treatments. Whether the residual materials were poisonous or not must also be considered. Additionally, although there have been some physical and chemical approaches for the removal of AFs, defects, including detoxification efficiency, high costs, health safety, and nutrient retention, still exist before large-scale application [13,14,15]. Consequently, biological degradation is considered a better alternative to the above methods, owing to its high specificity with harmless products. It overcomes their short board, thus making it the most practical method [1,16,17].

In general, the removal of AFs using biological methods consists of two methods, one of which is microbial adsorption based on the special structure of phosphoric acid and/or peptidoglycan of the microbial cell wall. This process mainly depends on hydrophobic and electrostatic interaction, which is typical of strains belonging to *Lactobacillaceae* [18,19,20,21] and *Saccharomyces* [22,23], etc. The other biological method for AF elimination usually refers to the degradation conducted by the microbial synthesis of enzymes and secondary metabolites which can convert the original structures of mycotoxins to non-toxic or less toxic constitutions. *Nocardia corynebacterioides* was first identified as an AFB_1_-degrading microorganism in 1966 [24]. Active substances secreted by microbes, such as *Bacillaceae* [25,26,27], *Staphylocococcus warneri*, *Sporosarcina* sp., *Lysinibacillus fusiformis* [28], *Enterococcus faecium* strains [29], *Pseudomonadaceae* [30,31], *Rhodococcus erythropolis* [32], and so forth, convert AFs to other atoxic substances without the coumarin lactone ring basic toxic structure in AFs [5]. Aflatoxin oxidase is a special intracellular degrading enzyme isolated from *Armillariella tabescens* [33,34,35] that interacts with the dilute ether bond of the furan ring in AFB_1_ and converts it to epoxide, which has less toxicity. However, the main AF-degrading substances currently known are secreted as extracellular enzymes, such as laccase [36,37], peroxidase [38], reductase [39], etc.

While these bacteria show the potential for AFB_1_ degradation, the species of *Microbacterium* genera have not been reported to possess this property for the moment. Moreover, few strains are broadly used in commercial applications, considering their narrow operation temperature range, relatively long incubation time and low degradation efficiency. In this study, a strain of *Microbacterium* genera from cow dung, which is successfully isolated using coumarin as the sole carbon source, demonstrates a high performance of AFB_1_ degradation at a broad working temperature range. The optimal degradation conditions and the cytotoxic potential of the degradation metabolites are also characterized.

## 2. Results

### 2.1. Isolation, Identification and Characterization of AFB_1_ Degradation Bacterial Strains

**Figure 1:**
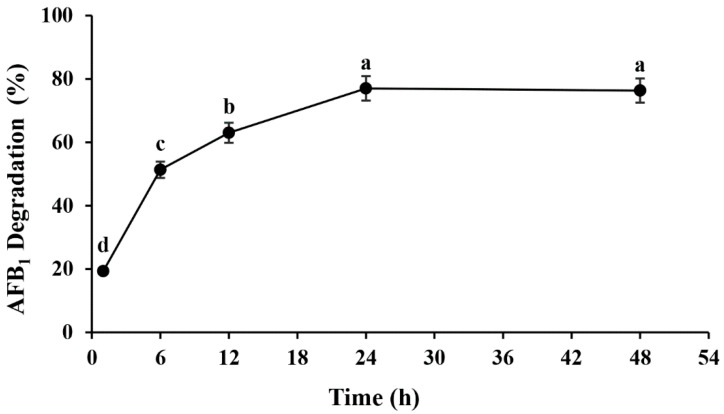
The detoxification effects on AFB_1_ by *M. proteolyticum* at different incubation times. Different letters indicate significant differences among the means according to Duncan test (*p* < 0.05).

AFs are a group of bisfuranocoumarin derivatives, and coumarin is the fundamental molecular structure of all AFs [40]. Thus, coumarin solid plates were applied as the sole carbon source for preliminary screening, followed by secondary screening using high-performance liquid chromatography (HPLC). Through two-round screening, *Microbacterium proteolyticum* strain B204, which showed the highest degradation activity (77.00 ± 1.53%) after 24 h of incubation at 30 °C (Figure 1) was then selected for further investigation. After incubation at 30 °C for 1 h, 19.33 ± 3.79% AFB_1_ was removed successfully, as shown in Figure 1. With the incubation time extended, the removal of AFB_1_ appeared to increase slowly, and the degradation ratio reached 77.00 ± 1.53% at 24 h. Conversely, the concentration of AFB_1_ in the control group remained stable from 0 to 24 h.

**Figure 2:**
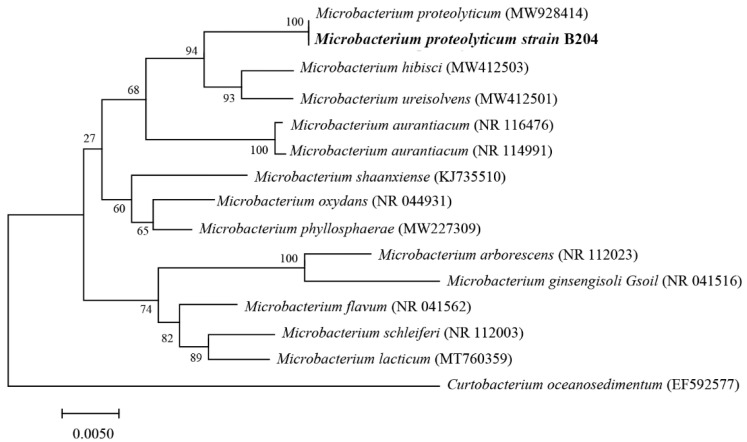
Phylogenetic relationship between *M. proteolyticum* strain B204 and other related species of the genus *Microbacterium*.

Based on the 16S rRNA gene sequence blast and phylogenetic evolution analysis, the *M. proteolyticum* strain B204 showed 100% nucleotide identity with *Microbacterium proteolyticum* (Figure 2). Morphological and biochemical analysis also confirmed the typical characteristics of *Microbacterium* sp., a Gram-positive, creamy yellow bacterium. This strain of *Microbacterium proteolyticum* was the first species in the *Microbacterium* genera discovered to possess the ability to degrade AFB_1_.

### 2.2. Effects of Incubation pH and Temperature on AFB_1_ Degradation by M. proteolyticum B204

**Figure 3:**
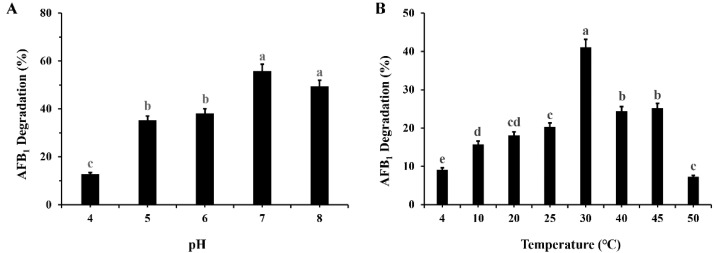
The influence of pH (**A**) and temperature (**B**) on the biodegradation of AFB_1_ after 12 h incubation with *M. proteolyticum* B204. Different letters indicate significant differences among the means according to the Duncan test (*p* < 0.05).

The influence of pH and temperature on AFB_1_ degradation by the *M. proteolyticum* strain B204 is presented in Figure 3. As we can see from Figure 3A, the strain *M. proteolyticum* B204 shows a broad working range of pH gradient between five and eight, and the degradation rate reaches up to 55.86 ± 2.73% at pH 7, while the ratios are similar at pH 5 and 6 (35.28 ± 5.90% and 38.16 ± 3.30%, respectively). Nevertheless, the minimum degradation efficiency fell to 12.77 ± 3.42% at pH 4.

It should be noted that the degradation of the AFB_1_ by strain *M. proteolyticum* B204 was barely affected by temperature (Figure 3B). The degradation rate remained almost stable at 15.78 ± 0.37%–20.31 ± 0.58% with increasing temperature up to 30 °C. The excellent thermostability suggests that strain 204 has terrific potential practical application.

### 2.3. AFB_1_ Degradation by Cell-Free Culture Supernatant and Cell Extracts of M. proteolyticum B204

**Figure 4:**
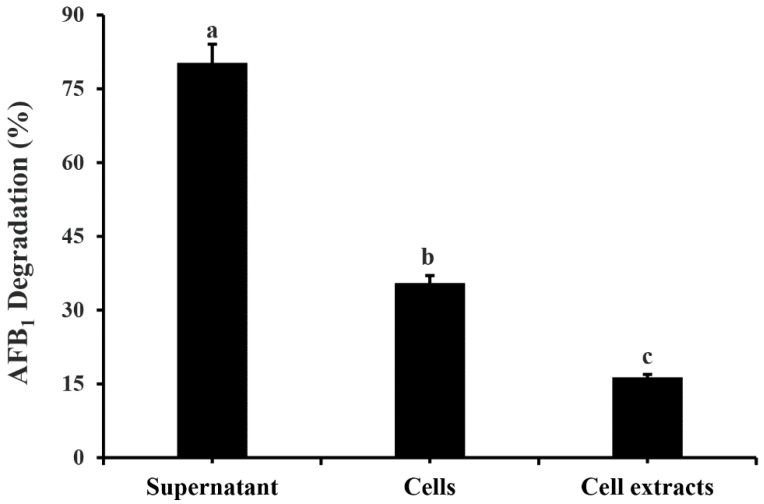
The degradation of AFB_1_ by cell-free supernatant and cell extracts after 24 h incubation at 30 °C. Different letters indicate significant differences among the means according to the Duncan test (*p* < 0.05).

As Figure 1 shows, the AFB_1_ degradation ratio reaches its peak after 24 h incubation. The cell-free culture supernatant of *M. proteolyticum* B204 exhibited the highest degradation ratio of 80.09 ± 1.29%, making it much more effective than cell extracts and cells, which degraded AFB_1_ at a ratio of 16.13 ± 2.95%, 35.28 ± 1.55%, respectively (Figure 4). This implies that the removal of AFB_1_ using *M. proteolyticum* B204 mainly depends on the biodegradation process, rather than the bio-adsorption to the bacterial cell wall. Accordingly, cell-free culture supernatant was then used in the follow-up experiments.

### 2.4. Effects of Heat Treatment, SDS, Proteinase K and EDTA on AFB_1_ Degradation by M. proteolyticum B204 Cell Culture Supernatant

**Figure 5:**
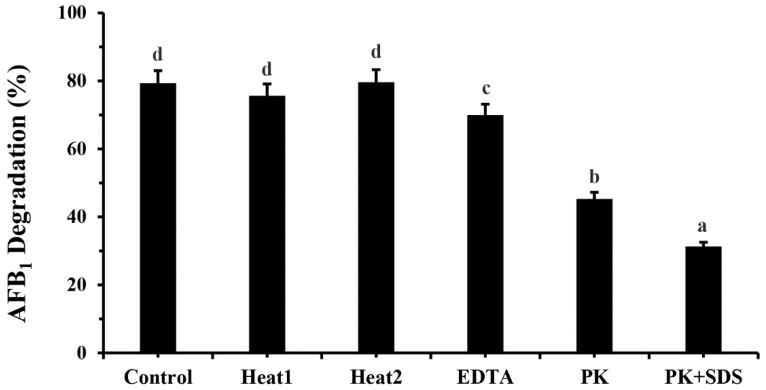
Degradation of AFB_1_ by heat-treated, EDTA-treated, proteinase K-treated and SDS plus proteinase K-treated cell-free supernatants of *M. proteolyticum* B204. Heat1: boiled for 30 min; Heat2: autoclaved for 30 min. PK: Proteinase K. Different letters indicate significant differences among the means according to Duncan test (*p* < 0.05).

The degradation rate remained stable even after boiling and sterilization at 75.33 ± 0.58% and 79.33 ± 1.53%, respectively, compared to the control group with the addition of culture broth without other treatments, demonstrating excellent thermostability. The detoxification efficiency (79.07 ± 1.94%) suffered little impact from the addition of EDTA, while this value was markedly affected by Proteinase K and Proteinase K plus SDS in the cell-free supernatant of strain *M. proteolyticum,* falling to 44.93 ± 3.68% and 31.21 ± 4.66%, respectively (Figure 5).

### 2.5. Cytotoxicity Analysis of M. proteolyticum B204

**Figure 6:**
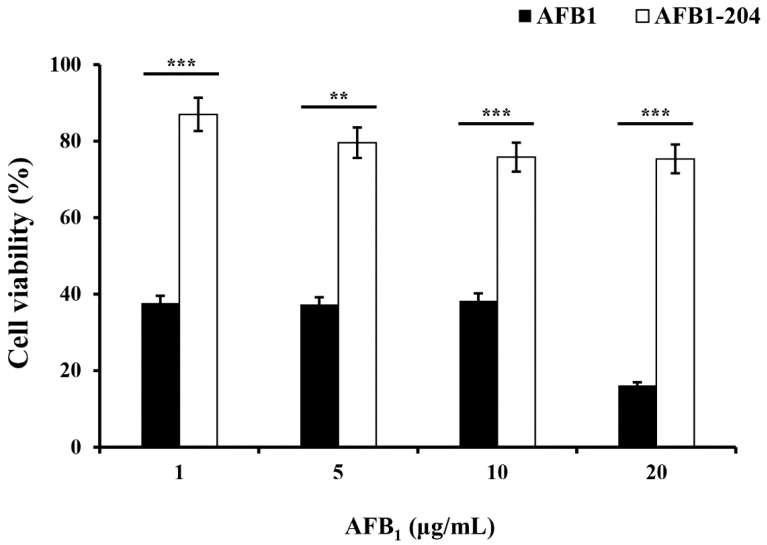
Analysis of AFB_1_ degradation products on cell viability by *M. proteolyticum* B204 cell-free supernatant after 24 h degradation. Results are described as means of three replicates and marked with standard errors. Means which are significantly different based on the *t*-test are indicated with asterisks (*p* < 0.05). ** *p* < 0.01; *** *p* < 0.001.

The potential toxicity and mutagenicity had to be considered, as some degradable products of toxins could still be toxic, similarly to their parent compounds. Concerns as to whether AFB_1_ degradation products formed by the strain *M. proteolyticum* B204 cell-free culture supernatant were more, less or not toxic are not unreasonable. Therefore, it is imperative to detect and analyze the toxicity of degradation products in comparison with AFB_1_ (the parent compound). The cytotoxicity of AFB_1_ degradation extracts was performed on human hepatocellular liver carcinoma (HepG2) cells by MTT assay in this study. The cell viability was relatively stable at around 37% with 1, 5 and 10 μg/mL AFB_1,_ until the concentration of AFB_1_ was raised to 20 μg/mL, which then decreased to 16.16% ± 2.42% (Figure 6). Delightfully, with the addition of cell-free culture supernatants of strain *M. proteolyticum* B204, cell mortality underwent an obvious loss. The cell viability was not less than 75% in every experimental group, which was much higher than in the control group, with the help of *M. proteolyticum* B204’s degradation of AFB_1_ (Figure 6). The cell viability was 86.99%, 79.59%, 75.82% and 75.36% with the addition of AFB_1_ at 1, 5, 10 and 20 μg/mL, respectively. These results indicate that the AFB_1_ degradation metabolites produced by strain *M. proteolyticum* B204 could help reduce the cytotoxicity AFB_1_ caused, and the degradation products themselves were harmless to cells as well.

### 2.6. Application on Food Matrices of AFB_1_ Detoxification by M. proteolyticum B204

**Figure 7:**
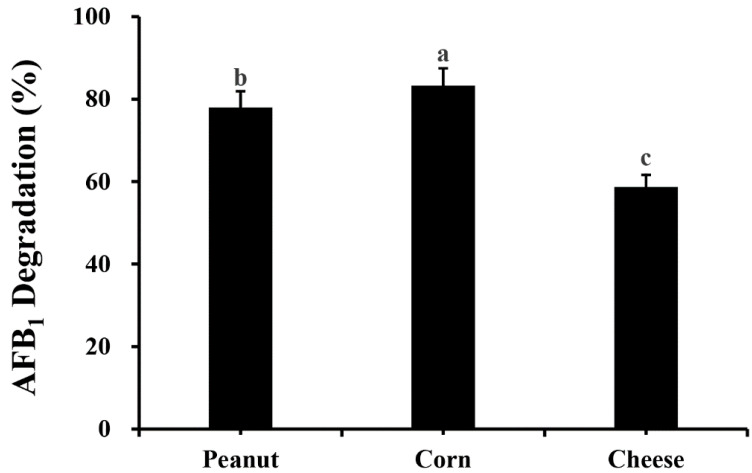
Application on food matrices of AFB_1_ degradation by *M. proteolyticum* B204 for 16 h. Different letters indicated significant differences among them according to Duncan test (*p* < 0.05).

Figure 7 shows that 78.00 ± 1.91%, 83.30 ± 1.35% and 58.69 ± 0.51% of AFB_1_ degradation occurred in peanuts, corn and cheese after incubation with strain *M. proteolyticum* B204 at 30 °C for 24 h. The most effective detoxification occurred in corn, while the worst was in cheese.

## 3. Discussion

Plenty of bacterial species have been revealed to be able to degrade AFB_1_ based on current research, including *Actinobacteria*, *Bacillus* and *α* or *β-proteobacteria* [1,41]. *Microbacterium proteolyticum* B204 isolated in our study is the first species ever reported in *Microbacterium* that exhibits AFB_1_ degradation ability, the degradation ratio of which was relatively high, reaching nearly 80% after 12 h treatment. However, this value did not exceed 90% in most bacteria. Among the bacteria with the highest degradation rate ever reported, *Escherichia coli* CG1061 eliminated 93.7% of AFB_1_ after 72 h incubation [42]. *Bacillus velezensis* DY3108 strains degraded up to 94.7% of AFB_1_ at 72 h, but only 30% after 12 h treatment. The use of *M. proteolyticum* B204 would greatly reduce time and cost and make this process more effective. Furthermore, the degradation process in our research was conducted with the incubation of bacteria and AFB_1_ solution together instead of the direct addition of substantial amounts of *M. proteolyticum* B204, which might have affected the degradation efficiency. There is currently no research that suggests that any species of *Microbacteriaceae* could degrade or adsorb AFB_1_. *Streptomyces roseolus* is the closest bacterial species to *M. proteolyticum* B204 that can remove AFB_1_ (Figure 2); it also shows strong inhibitory effects on aflatoxin production [43], suggesting that we could continue to study whether *M. proteolyticum* B204 has similar functions.

The degradation of AFB_1_ by cell-free supernatant after 24 h treatment was approximately 80% (Figure 3), almost equivalent to the effects induced by whole bacteria (Figure 1), but much higher than that of bacterial cell extracts (16.13%, Figure 3), indicating that the AFB_1_-degrading enzyme is mainly located in bacterial secretions. Similarly, it was also found that the cell-free supernatant of *Flavobacterium aurantiacum* could degrade 74.5% of AFB_1_ after 24 h incubation [44]; this AFB_1_-detoxification process primarily took place in the cell-free extracts of *Stenotrophomonas Maltophilia* 35-3 with a 78.7% degradation ratio after 72 h incubation with its culture supernatant [40]. Furthermore, the cell-free supernatant of *M. proteolyticum* B204 still possessed a AFB_1_ degradation capacity of more than 70% after treatment in boiling water at 100 °C for 20 min (Figure 5), implying its excellent thermal stability. We should also note that Proteinase K reduced almost 50% of the degradation activity and around 40% degradation still occurred. The reason why degradation kept proceeding mainly includes two aspects: firstly, there may have been more than one kind of enzyme that could degrade AFB_1_; secondly, the digestion of Proteinase K was not very specific, and partial resistance to Proteinase K might have also occurred.

As shown in Figure 6, AFB_1_ was cytotoxic to the growth of HepG2 cells, which has also been proved by previous studies [2,36,40,42,45]. Therefore, the AFB_1_ degradation ability of *M. proteolyticum* B204 was investigated by evaluating the remaining AFB_1_′s effects on cell proliferation and cell cytotoxicity by MTT assay. Based on the 86.99% cell viability in the 1 μg/mL AFB_1_ group instead of 100%, we presumed that the degradation ratio of B204 cell-free supernatant to AFB_1_ was about 80%, and it was the undegraded AFB_1_ that affected cell viability. As the concentration of AFB_1_ increased, the cell viability decreased, which might have been caused by the accumulation of residual AFB_1_ that had not been degraded.

## 4. Conclusions

Our study firstly reports that a strain of *Microbacterium proteolyticum* B204 from bovine faeces could degrade AFB_1_ effectively in common culture mediums and contaminated peanuts and corn with a ratio of approximately 77% after 24 h incubation. The toxicity evaluation of byproducts using the MTT assay on HepG2 cells showed a significant decrease in AFB_1_ after biodegradation, indicating its biosafety potential for actual use. These findings may offer a novel path for reducing AFB_1_ toxicity in practical use. Moreover, the effects of degradation products on human and animal metabolism should be further investigated, and the efficacy of isolated strains for detoxifying AFB_1_ may become of great interest for applications in food and feed.

## 5. Materials and Methods

### 5.1. Chemical and Medium

Luria-Bertani (LB) medium containing 10 g/L tryptone, 10 g/L NaCl and 5 g/L yeast extract was used for bacteria cultivation. Coumarin medium composed of 10 g/L coumarin, 0.5 g/L KH_2_PO_4_, 2 g/L NH_4_NO_3_, 0.002 g/L MgSO_4_ and 0.002 g/L FeSO_4_ was used for preliminary AFB1 degradation strain screening. AFB_1_ (C_17_H_12_O_6_) purchased from Yuanye Shengwu (ShanghaiyuanyeBio-Technology, Shanghai, China) was initially diluted in acetonitrile (HPLC grade) (MREDA, Beijing, China) to a stock solution of 100 mg/L, then filtered through a 0.22 μm filter (Millipore, New Jersey, USA), sealed in a brown bottle to avoid light preservation for better storage. Proteinase K was purchased from (Solarbio, Beijing, China). Metal salts and inorganic salts containing NaCl, MgSO_4_, FeSO_4_, FeCl_3_, KH_2_PO_4_, NH_4_NO_3_ and SDS powder were purchased from Solarbio (Beijing, China).

### 5.2. Two-Round Screening and Isolation of AFB_1_ Degradation Bacterium

Preliminary Screening: One gram of bovine faeces was dissolved in 10 mL sterilized distilled water after a continuous vortex for 3 min; it was then serially diluted to the final concentration of 10^−4^, 10^−5^ and 10^−6^. Aliquots of each diluent (100 μL) were homogeneously spread on coumarin medium plates and incubated at 30 °C for 21 days until visible colonies appeared. Single colonies growing well were picked and then inoculated to fresh new plates, the purification process of which was performed at least three times to obtain the pure isolates.

Secondary Screening: Each of the purified strains was further inoculated in 15 mL fresh LB liquid medium at 30 °C under continuous shaking of 180 rpm/min for 12–24 h. Fifty microliters of activated bacterial solution derived from the previous step were transferred to 9.95 mL fresh LB medium mixed with AFB_1_ solution to acquire the desired concentration of 10 μg/μL, then cultured in a rotary shaker incubator at 30 °C for 12 h away from light. The supernatant was collected after centrifugation at 8000 rpm for 20 min, followed by concentration and redissolution in 1 mL acetonitrile (HPLC grade), then filtered through 0.22 μm filter and preserved at 4 °C for the further detection of residual AFB_1_. AFB_1_ standard solution mixed with sterile LB medium (final concentration 10 μg/μL) was used as negative control.

### 5.3. Analysis of AFB_1_ Degradation Products Using HPLC

The prepared filtered samples were loaded on Waters C18 Column (0.5 μm, 4.6 × 250 mm) equipped with a fluorescence detector according to Guan et al., with some modifications [40]. Millipore water was used as mobile phase A, and a mixture with methanol and acetonitrile (*v/v*, 1:1) was employed as mobile phase B, with a flow rate of 0.6 mL/min. The concentration of residual AFB_1_ was quantitatively determined by fluorescence, the excitation wavelength and detection wavelength of which was set as 350 nm and 450 nm, respectively. AFB_1_ standard solution mixed with sterile LB medium instead of cell-culture supernatant was used as negative control. The AFB_1_ degradation ratio was evaluated using the following formula:

AFB_1_ degradation ration % = (AFB_1_ peak area in control group − AFB_1_ peak area in experimental group)/AFB_1_ peak area in control group × 100%

### 5.4. Identification of AFB_1_ Degradation Bacterium

Genomic DNA of *M. proteolyticum* B204 was extracted by TIANGEN bacterial DNA Kit (TIANGEN, Beijing, China) based on the manufacturer’s recommended instructions. Then, 16S rRNA gene fragments were amplified with Primer 27F (5′-AGAGTTTGATCCTGGCTCAG-3′) and Primer 1492R (5′-TACGGCTACCTTGTTACGACTT-3′). Afterwards, the sequence was aligned against known species with the NCBI BLAST algorithm (https://blast.ncbi.nlm.nih.gov, accessed on 29 July 2022) and EzTaxone BLAST analysis was undertaken (https://www.ezbiocloud.net, accessed on 29 July 2022, database version 07/07/2021) [46,47]. The phylogenetic tree was then constructed via the neighbor-joining method with the help of MEGA 6.0 version Software [48].

### 5.5. Exploration of Culture Conditions by M. proteolyticum B204 on AFB_1_ Degradation

The influences of different culture conditions on the biodegradation of AFB_1_ by *M. proteolyticum* B204 were achieved independently as follows: incubation time 12 h, initial culture pH 4, 5, 6, 7 and 8, incubation temperature 4, 10, 20, 25, 30, 40, 45 and 50 °C, with other conditions kept constant. The final concentration of AFB_1_ was consistent (10 μg/μL) and the addition of *M. proteolyticum* B204 was also maintained in each experimental group. The detection of AFB_1_ degradation efficiency was performed as described in Section 5.3 without modification.

### 5.6. AFB_1_ Degradation by Cell-Free Culture Supernatant, Cell-Free Extracts and Cells

The degradation of AFB_1_ by different components of *M. proteolyticum* B204 was investigated as described below, referring to Wang et al. with minor modifications [2]. Cell-free culture supernatant was collected after centrifugation at 8000 rpm for 20 min at 4 °C, followed by filtration through 0.22 μm filters and preserved on ice for a further AFB_1_ degradation assay. The precipitation of bacteria was thoroughly washed by sterilized MilliQ water and re-dissolved in PBS buffer (Biological Industries, Kibbutz Beit Haemek, Israel). The resuspending cells were then disrupted by ultrasonic homogenizer (Sonics, Wallingford, CT, USA) at 28% power with a 4 s pulse on and 6 s pulse off repetition cycle on ice. Cell-free extracts were obtained after centrifugation at 8000 rpm for 20 min at 4 °C and filtration through 0.22 μm filters. AFB_1_ standard solution was then introduced into cell-free culture supernatant, cell-free extracts and cells with a final concentration of 10 μg/μL, respectively. LB culture medium + PBS buffer with equivalent AFB_1_ was set as a negative control. After incubation at 30 °C for 24 h, the residual AFB_1_ was detected by HPLC as described in Section 5.3.

### 5.7. Effects of Heat Treatment, SDS, Proteinase and EDTA on AFB_1_ Degradation by Cell-Free Culture Supernatant

The effects of heat treatment, SDS, proteinase and EDTA on AFB_1_ degradation by *M. proteolyticum* B204 cell-free culture supernatant were studied according to Farzaneh et al., with slight adjustments [45]. The impact of heat treatment on AFB_1_ degradation efficiency was investigated by boiling for 20 min. Moreover, autoclave sterilization (121 °C, 20 min) was even adopted to evaluate the detoxification of AFB_1_. Sodium dodecyl sulfate (SDS) and Proteinase K treatments were also performed to evaluate the degradation process. One-milliliter of cell-free culture supernatant was mixed with 0.1 mL 10 mg/mL Proteinase K, 0.1 mL 10% SDS buffer and 0.1 mL 10 mg/mL Proteinase K plus 0.1 mL 10% SDS buffer, respectively. Moreover, EDTA with a final concentration of 0.1 mol/L was also added to 1 mL cell-free broth to analyze the degradation efficiency of AFB_1_. All the degradation reactions with the addition of SDS, Proteinase K and EDTA were incubated at 30 °C for 1 h in the dark. The degradation products of AFB_1_ were determined by HPLC as described in Section 5.3.

### 5.8. Cytotoxicity Analysis by MTT Assay

The effects of the AFB_1_ and B204 cell-free supernatant-induced degradation of AFB_1_ were detected on HepG2 cells via evaluating cell cytotoxicity using the MTT assay referenced in Wang et al., with some modifications [2]. The final concentrations of AFB_1_ were set as 0, 1, 5, 10 and 20 μg/mL, respectively. The only difference between experimental groups and control groups was the addition of *M. proteolyticum* B204 cell-free culture supernatant. After degradation for 24 h with the help of B204 cell-free culture supernatant, using equivalent LB culture medium as controls, the mixture was then incubated with HepG2 cells (1 × 10^4^ cells per well) in a 96-well multi-plate at 37 °C under 5% CO_2_ for 24 h. Then, 20 μL of 5 mg/mL MTT (3-(4,5-dimethylthiazol-2-yl)-2,5-diphenyltetrazolium bromide) was added and co-incubated for another 4 h. Finally, 150 μL DMSO was introduced to every well, making the cell crystals sufficiently dissolved. Cell viability was assessed according to the absorption value at 560 nm.

### 5.9. Application of AFB_1_ Detoxification by M. proteolyticum B204 to Food Matrices

The application to food matrices of *M. proteolyticum* B204 against AFB_1_ contamination was conducted on peanuts, corn and cheese. One gram of each sample mentioned above was sprayed with 100 μL AFB_1_ with a concentration of 100 μg/μL. After incubation with 450 μL of *M. proteolyticum* B204 at 30 °C for 16 h, the residual AFB_1_ was then detected by HPLC, as described above.

### 5.10. Statistical Analysis

All experiments were independently repeated in triplicate. The statistical analysis was performed by one-way analysis of variance (ANOVA) within the 95% confidence interval followed by the *Student*’s *t* test or *Duncan*’s test using SPSS software (SPSS Inc., Chicago, IL, USA).

## Data Availability

Not applicable.
